# The relationship between controlling nutritional status (CONUT) and cerebrovascular stenosis: a retrospective study with implications for ischemic stroke prevention

**DOI:** 10.7717/peerj.20968

**Published:** 2026-03-26

**Authors:** Ning Wang, Kun Guo, Bo Zhu, Yuanzhi Zhang, Xiaotao Jia, Zhiqin Liu, Zhengli Di, Naibing Gu, Ting Li

**Affiliations:** 1 The Third Affiliated Hospital of Xi’an Medical University, Xi’an, Shaanxi, China; 2 Department of Neurology, Tongchuan Mining Bureau Central Hospital, Tongchuan, Shaanxi Province, China; 3 Department of Neurology, Xi’an Central Hospital, Xi’an, Shaanxi Province, China; 4 Department of Neurology, Nuclear Industry 215 Hospital of Shaanxi Province/215 Hospital of Shaanxi Province, Xian Yan, Shaanxi, China; 5 Department of General Medicine, Xi’an Central Hospital, Xi’an, Shaanxi Province, China

**Keywords:** Ischemic stroke, CONUT score, Cerebral vascular stenosis, Correlation analysis

## Abstract

**Background:**

Ischemic stroke, a major global health concern, has high incidence, mortality, and disability rates. Cerebrovascular stenosis, a significant risk factor, is often assessed using digital subtraction angiography (DSA), which is invasive and costly. The Controlling Nutritional Status (CONUT) score, originally designed to assess malnutrition, has potential value in predicting vascular stenosis and stroke prognosis.

**Methods:**

This study analyzed 1,057 inpatients from Xi’an Central Hospital and Tongchuan Mining Bureau Central Hospital who underwent DSA for cerebrovascular stenosis from January 2016 to June 2024, comparing 674 patients with stenosis to 383 without. The CONUT score, calculated from serum albumin, lymphocyte count, and total cholesterol, was evaluated for its association with stenosis severity. Statistical analyses included t-tests, Mann-Whitney U tests, chi-square tests, Spearman correlation, stepwise regression, and ROC curve analysis, and age/comorbidity subgroup analyses.

**Results:**

CONUT scores were significantly higher in stenosis *vs.* controls (median 2 *vs.* 0, *p* < 0.001) and correlated with stenosis severity (Spearman’s *ρ* = 0.68, *p* < 0.001). Each 1-point CONUT increase elevated stenosis risk by 115% (OR = 2.15, 95% CI [1.89–2.47], *p* < 0.001), persisting after full adjustment for confounders. The score demonstrated moderate diagnostic accuracy (AUC = 0.794), with a sensitivity of 82.3% and a specificity of 76.1% at the optimal cutoff (CONUT score ≥ 5). outperforming traditional risk factors (age, diabetes, low density liproprotein cholesterol (LDL-C); all area under the curve (AUC) < 0.62). Subgroup analysis revealed enhanced predictive efficacy in adults >70 years (OR = 3.15) versus <50 years (OR = 1.82; interaction *p* = 0.012).

**Conclusion:**

The CONUT score is an independent predictor of cerebrovascular stenosis severity, with particular utility in elderly populations. It provides a practical, non-invasive tool for preliminary stenosis screening, potentially reducing unnecessary DSA referrals.

## Introduction

Ischemic stroke remains a major global health concern due to its high incidence, mortality, and disability rates (Campbell & Khatri, 2020). It severely affects people’s health and imposes a heavy burden on society, families, and patients. Atherosclerotic stenosis in intracranial and extracranial arteries increases stroke risk. Schmitzer et al. (2024) highlighted asymptomatic carotid artery stenosis (ICAS) as a primary risk factor for ischemic stroke, accounting for ∼10% of cases. It also leads to significant changes in patients’ brain structure and hemodynamics, which may indicate uneven capillary blood flow distribution, resulting in insufficient oxygen supply and reduced brain metabolism. Digital subtraction angiography (DSA) is considered the gold standard for diagnosing atherosclerotic stenosis of intracranial and extracranial arteries (Shaban et al., 2022). However, DSA is an invasive procedure, complex to perform, costly, and time-consuming. Therefore, there is a clinical need for a rapid assessment tool to evaluate the degree of cerebrovascular stenosis, assisting in preliminary screening and judgment, which is crucial for preventing ischemic stroke. The Controlling Nutritional Status (CONUT) score, developed by Ignacio de Ulíbarri et al. (2005), provides an objective, simple tool for malnutrition screening in hospitalized populations (Ignacio de Ulíbarri et al., 2005). It quantifies three serum biomarkers: albumin (reflecting protein reserves), total cholesterol (indicating caloric expenditure), and lymphocyte count (representing immune defence). Lower values of each component yield higher scores, with elevated totals indicating poorer nutritional status. Clinically, the CONUT score now extends beyond malnutrition screening. It serves as a prognostic indicator in heart failure, cancer, and ischemic stroke, offering a rapid assessment tool for cardiovascular and cancer risk stratification. Specifically, it predicts complications and mortality in oncology patients. Kato et al. (2020) observed that hospitalised patients with high CONUT scores faced increased risks of in-hospital mortality, non-cardiovascular death, and infections *versus* those with low scores. Notably, Naito et al. (2020) linked the score to stroke prognosis, while Field identified its utility in predicting 3-month post-stroke mortality. López Espuela et al., (2019) found that acute stroke patients with an admission CONUT score ≥5 had roughly double the 3-month mortality risk, confirming the score as an independent predictor of short-term post-stroke death. Conversely, Chen et al. (2023) reported lower CONUT scores in acute ischemic stroke (AIS) patients with hyperlipidemia.

In summary, the CONUT score demonstrates prognostic utility in cardiovascular disease and stroke. Given the shared mesodermal origin of cerebrovascular and cardiovascular systems—and their analogous angiogenic processes—alongside established links between cerebrovascular stenosis and stroke, we hypothesised a correlation between CONUT scores and cerebrovascular stenosis. Current evidence on this relationship remains limited. Our study therefore investigates whether CONUT scores correlate with cerebrovascular stenosis severity, aiming to provide a rapid preliminary screening tool to guide DSA referrals and inform stroke prevention strategies.

## Materials & Methods

### Study population

A total of 1,211 patients were screened; after applying the inclusion and exclusion criteria, 1,057 inpatients who underwent digital-subtraction angiography (DSA) at Xi’an Central Hospital and Tongchuan Mining Bureau Central Hospital between January 2016 and June 2024 were ultimately enrolled. Among them, 674 were confirmed to have intracranial arterial stenosis, while the remaining 383 served as non-stenosis controls.

### Inclusion criteria

#### Patients were eligible for inclusion if they met all of the following criteria

(1) Aged between 18 and 80 years.
(2) Underwent digital subtraction angiography (DSA) for suspected cerebrovascular ischemia (*e.g.*, transient ischemic attack (TIA), stroke, or focal neurological deficits) or for pre-intervention assessment of known stenosis.
(3) Had complete laboratory data within 24 h of admission necessary for calculating the CONUT score (serum albumin, lymphocyte count, and total cholesterol).
(4) Had undergone the DSA procedure with routine written informed consent obtained as part of standard clinical care.

#### Definition of groups for analysis

(1) Stenosis Group: Patients from the enrolled cohort with DSA-confirmed intracranial or extracranial arterial stenosis (≥50%).
(2) Control Group: Patients from the same enrolled cohort with DSA-confirmed absence of significant stenosis (<50%).

### Exclusion criteria

#### Patients meeting any of the following criteria were excluded from the study

(1) Severe cognitive impairment or any condition precluding comprehension of the study or provision of informed consent.
(2) History of major surgery or significant trauma within the past 3 months.
(3) Terminal illness with a life expectancy of less than 6 months, pregnancy, or lactation.
(4) Severe chronic liver disease (*e.g.*, cirrhosis), severe chronic kidney disease (*e.g.*, end-stage renal disease), active inflammatory or autoimmune diseases, or active cancer.
(5) A history of ischemic stroke, intracerebral hemorrhage, or a rapidly deteriorating neurological condition within the past 3 months, or prior DSA-confirmed cerebrovascular stenosis (to avoid confounding the baseline assessment).
(6) Incomplete clinical or laboratory data required for the study analysis.

### Data collection

The experimental data for this study were derived from the medical record management system of Xi’an Central Hospital and Tongchuan Mining Bureau Central Hospital. Covering basic information such as patients’ gender, age, medical history, alcohol and smoking history, and BMI values. Additionally, the data included laboratory test results within 24 h of admission, such as complete blood count (hemoglobin, red blood cells, platelets, neutrophils, monocytes, and lymphocytes), liver function (albumin, alanine aminotransferase), fasting blood glucose, glycated albumin, renal function (uric acid, creatinine), lipid profile (triglycerides, low-density lipoprotein, total cholesterol), systolic and diastolic blood pressure, and coagulation function.

### Ethics approval

This retrospective study was conducted in accordance with the Declaration of Helsinki and approved by the Institutional Review Boards (IRB)/Ethics Committees of both participating hospitals: Xi’an Central Hospital (Approval No: KY-2024-35) and Tongchuan Mining Beaural Hospital (Approval No: TCMBCH-2024-0005). The need for additional informed consent for this retrospective data analysis was waived by the IRBs, as the study involved anonymized data extracted from routine clinical records. All patients had provided written informed consent for the diagnostic DSA procedure as part of their standard clinical care.

### Scoring and assessment methods

#### CONUT score calculation

The CONUT score is calculated based on total lymphocyte count, serum total cholesterol levels, and serum albumin. The detailed scoring criteria are described in Table 1. For serum albumin, 0 points for levels of 35.0 g/L and above, 2 points for 30.0–34.0 g/L, 4 points for 25.0–29.0 g/L, and 6 points for below 25.0 g/L. For total lymphocyte count, 0 points for 1,600/ml and above, 1 point for 1,200–1,599/ml, 2 points for 800–1,199/ml, and 3 points for below 800/ml. Regarding serum total cholesterol, 0 points for levels of 4.65 mmol/L and above, 1 point for 3.62−4.64 mmol/L, 2 points for 2.58−3.61 mmol/L, and three points for below 2.58 mmol/L. The CONUT score is obtained by summing the above points. Nutritional status is categorized as follows based on the score: 0–1 indicates no malnutrition, 2–4 indicates mild malnutrition, 5–8 indicates moderate malnutrition, and 9–12 indicates severe malnutrition.

**Table 1:** CONUT score components and malnutrition classification.

**Biomarker**	**Threshold**	**Points**
Serum Albumin (g/L)	≥35.0	0
	30.0–34.9	2
	25.0–29.9	4
	<25.0	6
Lymphocyte Count (×10^6^/L)	≥1.60	0
	1.20–1.59	1
	0.80–1.19	2
	<0.80	3
Total Cholesterol (mmol/L)	≥4.65	0
	3.62–4.64	1
	2.58–3.61	2
	<2.58	3

**Notes.**

CONUT Controlling Nutritional Status Score sum of points from all three biomarkers

The CONUT Score assesses malnutrition severity: a score of 0–1 indicates normal nutritional status, 2–4 indicates mild malnutrition, 5–8 indicates moderate malnutrition, and 9–12 indicates severe malnutrition.

#### DSA procedure

**Preparation**: Evaluate patient, review data, perform exam, complete tests, and obtain consent.

**Process**: Puncture 1.5−2.0 cm below right femoral artery pulsation. Disinfect from umbilicus to mid-right thigh. Anesthetize with 2% lidocaine. Incise and puncture at 45 degrees. Insert guidewire after successful puncture. Place sheath-sleeve assembly. Confirm blood flow and flush with heparinized saline.

**Assessment Scope**: Aortic arch, subclavian, vertebral, common and internal carotid arteries. Extracranial: V1–V3 of subclavian and vertebral, C1–C5 of common and internal carotid. Intracranial: C6–C7 of internal carotid, M1-M2 of middle cerebral, A1–A2 of anterior cerebral, P1–P2 of posterior cerebral, V4 of vertebral, and basilar artery.

### Assessment of intracranial and extracranial vascular stenosis

Intracranial vascular stenosis severity was quantified using the Warfarin-Aspirin Symptomatic Intracranial Disease (WASID) trial methodology (Chimowitz et al., 2005). Extracranial stenosis assessment employed the North American Symptomatic Carotid Endarterectomy Trial (NASCET) criteria (Barnett et al., 1998). All DSA datasets underwent blinded independent evaluation by two neurointerventional radiologists (≥5 years’ DSA interpretation experience), with discordant cases adjudicated by a third senior specialist.

### Statistical analysis

Statistical analyses were performed following the methodology described by Guo et al. (2025). Briefly, we used Python 3.12.1, R 4.4.1, and IBM SPSS 28.0.0 (IBM Corp., Armonk, NY, USA) for data processing. Normality of data was assessed using the Kolmogorov–Smirnov test. Continuous variables with normal distribution were compared using independent samples *t*-tests, while non-normally distributed variables were analyzed with the Mann–Whitney *U* test. Categorical variables were evaluated using chi-square tests or Fisher’s exact tests as appropriate. Patients were grouped based on the degree of cerebrovascular stenosis to further explore the association between the CONUT score and the degree of cerebrovascular stenosis. Spearman correlation analysis was used to determine the correlation between the CONUT score and the degree of cerebrovascular stenosis, and a correlation heatmap was used for visualization. After adjusting for potential confounding variables, stepwise regression analysis was further employed to explore the relationship between the CONUT score and the degree of cerebrovascular stenosis. Additionally, the ability of the CONUT score to predict stenosis distribution was assessed, and a forest plot was used for visualization. Finally, odds ratios (OR) and 95% confidence intervals (CI) were calculated, and receiver operating characteristic (ROC) curves were plotted to determine the area under the curve (AUC) to assess diagnostic performance. To address potential effect modification, we conducted predefined subgroup analyses stratified by: Age groups, Comorbidity status. Interaction terms (CONUT × subgroup) were tested using likelihood ratio tests. Stratum-specific odds ratios and 95% confidence intervals were calculated *via* multivariate logistic regression adjusted for Model covariates. A two-tailed *p*-value of less than 0.05 was considered statistically significant.

## Results

### Baseline data of the comparison between stenosis group and control group

Significant differences in baseline characteristics were identified between the stenosis group (*n* = 674) and the control group (*n* = 383), as is show in Table 2. Specifically, the average age of the stenosis group was slightly higher than that of the control group (54.9 years *vs.* 54.0 years, *p* = 0.04), In terms of lifestyle, the proportion of smokers in the stenosis group (26.0%) was significantly higher than in the control group (15.4%, *p* < 0.001), and the proportion of alcohol consumers was also higher (16.0% *vs.* 9.4%, *p* = 0.003). Regarding physiological indicators, the diastolic blood pressure of the stenosis group (76.0 mmHg) was slightly higher than that of the control group (75.3 mmHg, *p* = 0.045), and the heart rate was significantly faster (70.2 bpm *vs.* 69.1 bpm, *p* < 0.001). In terms of blood lipids, the total cholesterol level of the control group (5.0 mmol/L) was higher than that of the stenosis group (4.7 mmol/L, *p* < 0.001), while the difference in fasting blood glucose levels was not significant (*p* = 0.081). The albumin level of the stenosis group (37.7 g/L) was significantly lower than that of the control group (40.5 g/L, *p* < 0.001), and the lymphocyte count was also slightly lower (1.9 ×10^9^/L *vs.* 2.0 ×10^9^/L, *p* = 0.007). Additionally, the CONUT score (median 2) of the stenosis group was significantly higher than that of the control group (median 0, *p* < 0.001).

**Table 2:** Comparison of baseline characteristics and CONUT scores between the control group and stenosis group in cerebrovascular stenosis.

**Factors**	**Control group**	**Stenosis group**	**t/Z**	***p* value**
	**(*n* = 383)**	**(*n* = 674)**		
**Male, n (%)**	240 (62.7)	430 (63.8)	0.136	0.713
**Age, years**	54.0 ± 4.4	54.9 ± 4.5	−2.881	0.04
**Hypertension, n (%)**	166 (43.3)	330 (49.0)	3.096	0.078
**Diabetes, n (%)**	70 (18.3)	168 (24.9)	6.189	0.013
**Smoker, n (%)**	59 (15.4)	175 (26.0)	15.799	<0.001
**History of drinking, n (%)**	36 (9.4)	108 (16.0)	9.107	0.003
**BMI, Kg/m^2^**	23.0 ± 2.1	23.0 ± 1.9	−0.267	0.79
**SBP, mmHg**	137.6 ± 10.7	138.1 ± 10.2	−0.732	0.465
**DBP, mmHg**	75.3 ± 5.1	76.0 ± 5.0	−2.005	0.045
**HR, bpm**	69.1 ± 3.8	70.2 ± 5.1	−3.848	<0.001
Lymphocyte, 10*9/L	2.0 ± 0.6	1.9 ± 0.7	2.704	0.007
**Albumin, g/L**	40.5 ± 4.3	37.7 ± 6.2	7.629	<0.001
**Total cholesterol, mmol/L**	5.0 ± 0.8	4.7 ± 1.1	5.082	<0.001
**FBP, mmol/L**	6.12 ± 2.11	6.37 ± 2.30	−1.747	0.081
**Triglycerides, mmol/L**	1.64 ± 0.12	1.68 ± 0.13	−0.678	0.498
**Glycated serum albumin**	15.1 ± 3.2	15.4 ± 4.2	−1.006	0.314
**LDL cholesterol, mmol/L**	2.68 ± 0.98	2.66 ± 0.92	0.22	0.826
**HDL cholesterol, mmol/L**	1.33 ± 0.49	1.27 ± 0.53	1.837	0.066
**WBC, 1,012/L**	6.2 ± 1.8	6.4 ± 2.0	−1.052	0.293
**RBC, 1,012/L**	4.6 ± 0.6	4.6 ± 0.5	0.158	0.874
**HGB, g/L**	137.8 ± 19.3	137.3 ± 19.2	0.392	0.695
**Platelet, 109/L**	207.8 ± 60.6	204.8 ± 60.7	0.787	0.431
**Creatinine, μmol/L**	75.1 ± 45.6	74.6 ± 42.3	0.171	0.865
**Uric acid, μmol/L**	367.7 ± 114.3	358.9 ± 111.2	1.215	0.225
**LVEF, %**	54.8 ± 7.4	53.7 ± 7.2	2.455	0.014
**Prothrombin time**	11.7 ± 3.4	11.7 ± 3.4	0.2	0.814
**Fibrinogen quantitation**	3.17 ± 0.8	3.11 ± 0.7	1.143	0.253
**FDP**	2.83 ± 0.81	3.11 ± 0.72	−0.702	0.483
**Alanine aminotransferase**	23.1 ± 5.92	25.3 ± 6.32	−1.312	0.19
**Polymorphonuclear neutrophils**	3.82 ± 1.49	3.94 ± 1.68	−1.111	0.267
**MON**	0.38 ± 0.14	0.39 ± 0.15	−1.32	0.187
**CONUT score**	0 (0,1)	2 (0,4)	−16.702	<0.001

**Table 3:** Comparison of clinical and biochemical parameters among different stenosis groups.

**Factors**	**Control group** **(*n* = 383)**	**Mild stenosis** **(*n* = 290)**	**Moderate stenosis** **(*n* = 211)**	**Severe Stenosis** **(*n* = 173)**	***p* value**	***p*∗ < 0.05**
**Male, n (%)**	240 (62.7)	189 (65.2)	135 (64.0)	106 (61.3)	0.322	
**Age, years**	54.0 ± 4.4	54.3 ± 4.2	55.1 ± 5.0	55.5 ± 4.5	<0.001	^b^ ^,^ ^c^ ^,^ ^e^
**Hypertension, n (%)**	166 (43.3)	138 (47.6)	105 (49.8)	87 (50.3)	0.322	
**Diabetes, n (%)**	70 (18.3)	71 (24.5)	60 (28.4)	37 (21.4)	0.03	^b^
**Smoker, n (%)**	59 (15.4)	60 (20.7)	58 (27.5)	57 (32.9)	<0.001	^b^ ^,^ ^c^ ^,^ ^e^
**History of drinking n (%)**	36 (9.4)	55 (19.0)	32 (15.2)	21 (12.1)	0.004	^a^ ^,^ ^b^
**BMI, Kg/m2**	23.0 ± 2.1	23.1 ± 1.9	23.2 ± 2.0	22.8 ± 1.8	0.175	^f^
**SBP, mmHg**	137.6 ± 10.7	138.1 ± 10.0	138.7 ± 10.2	137.3 ± 10.6	0.502	
**DBP, mmHg**	75.3 ± 5.1	76.2 ± 5.2	75.8 ± 4.7	75.8 ± 5.0	0.184	^a^
**HR, bpm**	69.1 ± 3.8	70.5 ± 5.1	69.8 ± 4.6	70.4 ± 5.6	0.001	^a^ ^,^ ^c^
Lymphocyte, 10*9/L	2.04 ± 0.58	2.04 ± 0.55	1.92 ± 0.70	1.78 ± 0.78	<0.001	^b^ ^,^ ^c^ ^,^ ^d^ ^,^ ^e^ ^,^ ^f^
**Albumin, g/L**	40.5 ± 4.3	38.7 ± 4.9	38.2 ± 6.1	35.7 ± 7.6	<0.001	^a^ ^,^ ^b^ ^,^ ^c^ ^,^ ^e^ ^,^ ^f^
**Total cholesterol, mmol/L**	5.0 ± 0.8	4.9 ± 0.9	4.6 ± 1.2	4.4 ± 1.2	<0.001	^b^ ^,^ ^c^ ^,^ ^e^ ^,^ ^f^
**FBP, mmol/L**	6.12 ± 2.15	6.23 ± 2.53	6.00 ± 2.03	7.07 ± 2.05	<0.001	^c^ ^,^ ^e^ ^,^ ^f^
**Triglycerides, mmol/L**	1.64 ± 0.12	1.56 ± 0.74	1.70 ± 0.24	1.89 ± 0.13	0.021	^c^ ^,^ ^e^
**Glycated serum albumin**	15.1 ± 3.2	15.5 ± 4.3	15.2 ± 3.4	15.5 ± 4.90	0.591	
**LDL cholesterol, mmol/L**	2.68 ± 0.98	2.59 ± 0.88	2.74 ± 0.98	2.69 ± 0.92	0.347	
**HDL cholesterol, mmol/L**	1.33 ± 0.49	1.27 ± 0.47	1.35 ± 0.69	1.17 ± 0.36	0.002	^c^ ^,^ ^e^ ^,^ ^f^
**WBC, 1012/L**	6.24 ± 1.81	6.35 ± 1.88	6.43 ± 2.09	6.33 ± 1.75	0.71	
**RBC, 1012/L**	4.56 ± 0.56	4.53 ± 0.58	4.58 ± 0.63	4.56 ± 0.54	0.802	
**HGB, g/L**	137.8 ± 19.3	136.9 ± 21.3	137.5 ± 18.3	138.0 ± 16.5	0.909	
**Platelet, 109/L**	207.9 ± 60.3	201.8 ± 61.9	206.9 ± 62.6	207.3 ± 56.5	0.595	
**Creatinine, μmol/L**	75.1 ± 45.6	74.5 ± 22.4	74.2 ± 34.9	75.3 ± 36.2	0.992	
**Uric acid, μmol/L**	367.7 ± 114.3	357.7 ± 112.0	364.4 ± 117.9	354.3 ± 101.5	0.511	
**LVEF, %**	54.8 ± 7.4	53.8 ± 7.0	54.4 ± 7.3	52.5 ± 7.4	0.005	^c^ ^,^ ^f^
**Prothrombin time**	11.7 ± 3.4	11.7 ± 3.9	11.8 ± 3.3	11.4 ± 2.3	0.667	
**Quantification of fibrinogen**	3.16 ± 0.79	3.11 ± 0.76	3.11 ± 0.74	3.11 ± 0.74	0.727	
**Fibrin degradation products**	2.83 ± 0.80	3.22 ± 0.55	2.99 ± 0.36	3.07 ± 0.33	0.881	
**Alanine aminotransferase**	23.1 ± 5.98	23.8 ± 6.87	23.2 ± 5.49	23.8 ± 5.66	0.785	
**Neutrophil count**	3.82 ± 1.49	3.91 ± 1.58	4.00 ± 1.93	3.91 ± 1.51	0.644	
**The number of monocytes**	0.38 ± 0.14	0.39 ± 0.15	0.40 ± 0.15	0.39 ± 0.15	0.565	
**CONUT score**	0 (0,0)	1 (0,2)	3 (1,4)	4 (2,6)	<0.001	^a^ ^,^ ^b^ ^,^ ^c^ ^,^ ^e^ ^,^ ^f^

**Notes.**

The meanings of the superscript letters a–f in Table 3 footnotes are as follows:

a indicates a significant difference between the control group and the mild stenosis group.

b indicates a significant difference between the control group and the moderate stenosis group.

c indicates a significant difference between the control group and the severe stenosis group.

d indicates a significant difference between the mild and moderate stenosis groups.

e indicates a significant difference between the mild and severe stenosis groups.

f indicates a significant difference between the moderate and severe stenosis groups.

When multiple letters appear beside a variable, all corresponding group pairs show significant differences. A single letter denotes significance only for that specific pair. Absence of letters indicates no significant intergroup differences were detected in post hoc comparisons, even if the overall *p*-value was < 0.05.

### Analysis of baseline clinical data across different stenosis severity groups (Table 3)

Baseline characteristics across control, mild, moderate, and severe stenosis groups are presented in Table 3. Notable differences included: no significant difference in gender distribution (*p* > 0.05); a significant age difference with the severe stenosis group being oldest (*p* < 0.001); no significant variation in hypertension but increased diabetes prevalence; a rise in smoking and drinking with stenosis severity (*p* < 0.001 or *p* < 0.05); no significant BMI changes (*p* > 0.05); stable blood pressure and heart rate (*p* > 0.05); significant differences in lymphocyte count, albumin, total cholesterol, FBP, and HDL cholesterol (*p* < 0.001 or *p* < 0.05); and no significant differences in hemoglobin, platelet count, creatinine, uric acid, left ventricular ejection fraction, or prothrombin time (*p* > 0.05).

### Analysis of AUC values and correlation heatmap

#### AUC value analysis

**Figure 1:**
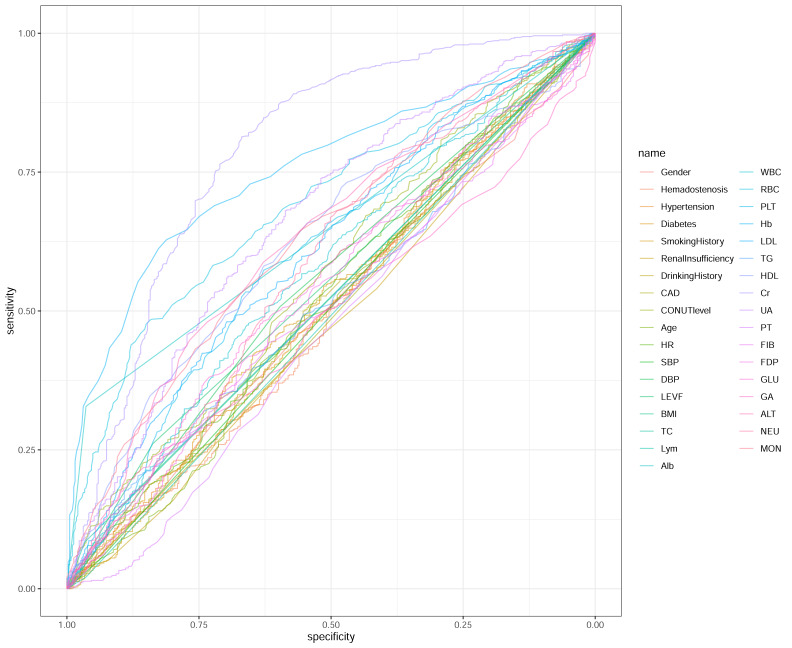
Predictive performance assessment of clinical biomarkers for cerebrovascular stenosis.

The CONUT composite score exhibited the best discriminative capacity (AUC = 0.794) for cerebrovascular risk stratification, outperforming its individual components: albumin (AUC = 0.73), lymphocyte count (AUC = 0.71), and total cholesterol (AUC = 0.68). Other biomarkers with moderate discriminative ability included creatinine (AUC = 0.789) and hemoglobin (AUC = 0.755). The ROC curves for all variables are detailed in Fig. 1. (AUC interpretation: 0.7−0.8 = moderate; 0.6−0.7 = limited; <0.6 = negligible.)

#### Correlation heatmap analysis

**Figure 2:**
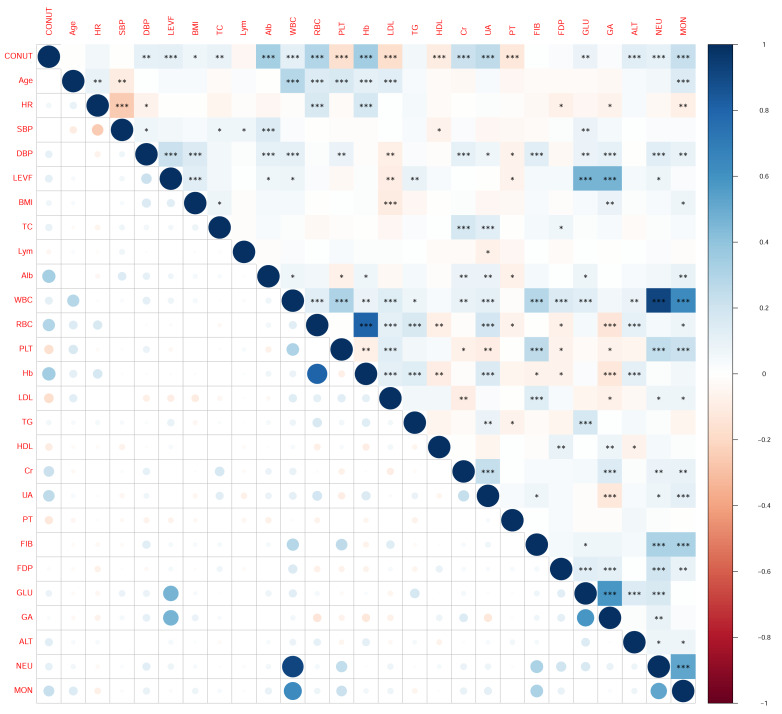
Associations between CONUT score and clinical variables assessed by Spearman’s correlation analysis.

As illustrated in Fig. 2, hierarchical clustering of Spearman correlations revealed three distinct patterns: (1) significant positive associations between CONUT score and nutritional biomarkers, most notably with albumin (*ρ* = 0.82, *p* < 0.001) and lymphocyte count (*ρ* = 0.79, *p* < 0.001); (2) inverse correlations with inflammatory markers, particularly C-reactive protein (*ρ*=−0.63, *p* < 0.001) and neutrophil count (*ρ*=−0.58, *p* < 0.001); and (3) a CONUT-centric clustering pattern in the upper-left quadrant indicating its pivotal role in the correlation network. These associations remained significant after FDR correction (*q* < 0.01), with the nutritional-inflammation axis accounting for 68% of the observed variance.

### Stepwise regression analysis of CONUT predictive power for cerebrovascular stenosis

The association between the CONUT score and cerebrovascular stenosis was evaluated using four multivariate regression models with stepwise adjustment for confounders are summarized in Table 4. All models demonstrated a significant relationship between the CONUT score and stenosis risk, with risk increasing alongside the score. Model 1 (adjusted for sex, age, heart rate (HR), systolic blood pressure (SBP), diastolic blood pressure (DBP)): OR = 2.14 per 1-point increase in CONUT; ORs for Q2, Q3, and Q4 *versus* Q1 were 7.0, 57.3, and 161.6, respectively (all *p* < 0.001). Model 2 (additionally adjusted for ejection fraction (EF), body mass index (BMI), hypertension, diabetes, alcohol history, renal insufficiency): OR = 2.15; ORs for Q2–Q4 were 7.1, 60.4, and 175.8, respectively (all *p* < 0.001). Model 3 (further adjusted for smoking history, WBC, RBC, platelet count, low density lipoprotein-cholesterol (LDL-C), triglycerides): OR = 2.16; ORs for Q2–Q4 were 7.2, 59.3, and 177.7, respectively (all *p* < 0.001). Model 4 (additionally adjusted for High-Density Lipoprotein Cholesterol (HDL-C), Creatinine (Cr), Uric Acid (UA), Prothrombin Time (PT), Fasting Blood Glucose (FBG), Fibrinogen (FIB), Fibrinogen Degradation Products (FDP), Alanine Aminotransferase (ALT), neutrophils, monocytes): OR = 2.15; ORs for Q2–Q4 were 7.3, 60.7, and 184.0, respectively (all *p* < 0.001). To sum up, Each 1-unit increase in CONUT score consistently elevated risk by ∼115% (OR ≈ 2.15, 95% CI [1.89–2.47], *p* < 0.001); When stratified by quartiles (Q1 as reference): Q2 group showed a 7-fold risk increase (OR: 7.0–7.3), Q3 group surged to 60-fold (OR: 57.3–60.7), Q4 group peaked at 184-fold (OR: 161.6–184.0), with all comparisons *p* < 0.001. This association remained robust across models—from baseline adjustments (demographics/blood pressure) to fully adjusted models (metabolic/coagulation/inflammatory markers)—exhibiting minimal variation in ORs (<1%). These results confirm the CONUT score as a strong independent predictor of cerebrovascular stenosis.

**Table 4:** Stepwise regression analysis of the CONUT predictive power for cerebrovascular sclerosis: odds ratios of multiple models.

		**Conut score** **Cerebrovascular Stenosis**		
		**OR**	**95% CI**	***p*** ** value**
**Model 1**	**Continuous variable**	2.143	1.893–2.426	<0.001
	**Per quartile**	7.008	5.590–8.785	<0.001
	**Q1**	–	–	
	**Q2**	57.274	30.328–108.161	<0.001
	**Q3**	161.645	81.960–318.802	<0.001
	**Q4**	376.839	174.649–356.20	<0.001
**Model 2**	**Continuous variable**	2.154	1.900–2.442	<0.001
	**Per quartile**	7.107	5.646–8.947	<0.001
	**Q1**	–	–	
	**Q2**	60.390	31.505–115.760	<0.001
	**Q3**	175.767	87.539–352.919	<0.001
	**Q4**	412.366	187.770–905.604	<0.001
**Model 3**	**Continuous variable**	2.155	1.899–2.47	<0.001
	**Per quartile**	7.199	5.701–9.091	<0.001
	**Q1**	–	–	
	**Q2**	59.319	30.841–114.092	<0.001
	**Q3**	177.727	88.001–358.939	<0.001
	**Q4**	410.624	186.358–904.775	<0.001
**Model 4**	**Continuous variable**	2.149	1.891–2.441	<0.001
	**Per quartile**	7.257	5.727–9.196	<0.001
	**Q1**	–	–	
	**Q2**	60.689	31.392–117.327	<0.001
	**Q3**	184.043	90.275–375.209	<0.001
	**Q4**	409.980	184.640–910.330	<0.001

**Notes.**

Model 1: Adjusted for gender, age, HR, SBP, and DBP.

Model 2: Adjusted for gender, age, HR, SBP, DBP, EF, BMI, hypertension, diabetes, alcohol history, and renal insufficiency.

Model 3: Adjusted for gender, age, HR, SBP, DBP, EF, BMI, hypertension, diabetes, alcohol history, renal insufficiency, smoking history, WBC, RBC, platelets, LDL-C, and triglycerides.

Model 4: Adjusted for gender, age, HR, SBP, DBP, EF, BMI, HTN, DM, alcohol history, renal insufficiency, smoking history, WBC, RBC, PLT, LDL-C, TG, HDL-C, Cr, UA, PT, FBG, FIB, FDP, GSA, ALT, neutrophils, and monocytes.

### Subgroup analyses by age and comorbidity

The predictive efficacy of the CONUT score showed a statistically significant age-dependent interaction (P for interaction = 0.012) as shown in Table 5. Specifically, in individuals aged > 70 years, each 1-point increment in the CONUT score was associated with a 215% significantly elevated risk of vascular stenosis (odds ratio (OR) = 3.15, 95% confidence interval (CI) [2.38–4.17]). Conversely, among those aged < 50 years, the same magnitude of CONUT score increase resulted in only an 82% risk elevation (OR = 1.82). Notably, comorbidity status did not significantly alter the predictive value of the CONUT score: in the diabetes subgroup (interaction *p* = 0.32), the OR was 2.25 (95% CI [1.82–2.78]); in the hypertension subgroup (interaction *p* = 0.41), the OR was 2.18 (95% CI [1.88–2.53]). Both comorbidity subgroups showed no statistically significant differences in risk estimates compared to the comorbidity-free reference group (OR = 2.11; all interaction *p* > 0.05).

**Table 5:** Odds ratio of the association between CONUT score and cerebrovascular stenosis risk in subgroup analysis.

Subgroup	each 1-point increase in CONUT, the odds ratio (95% CI)	*p*-interaction
Age Stratification		0.012
<50 years (*n* = 214)	1.82 (1.31–2.53)	
50–70 years (*n* = 638)	2.20 (1.91–2.54)	
>70 years (*n* = 205)	3.15 (2.38–4.17)	
Comorbidity Status		
Diabetes (*n* = 297)	2.25 (1.82–2.78)	0.32
Hypertension (*n* = 586)	2.18 (1.88–2.53)	0.41
No Comorbidity (*n* = 174)	2.11 (1.65–2.70)	Reference

### ROC curve analysis: predictive ability of the conut score for cerebral vascular stenosis

**Figure 3:**
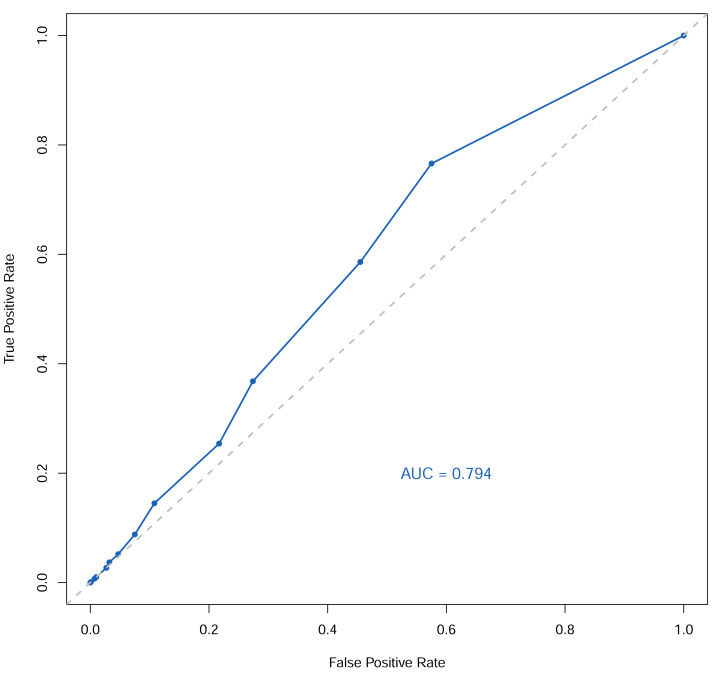
ROC curve for CONUT score in predicting cerebrovascular stenosis.

At the optimal cutoff (score ≥5), the CONUT score demonstrated moderate discriminative capacity for cerebrovascular stenosis (AUC = 0.794, sensitivity 82.3%, specificity 76.1%) as shown in Fig. 3. Its performance was superior to albumin (ΔAUC = 0.064) and lymphocyte count (ΔAUC = 0.084), but substantially below imaging gold standards (Digital Subtraction Angiography (DSA) AUC > 0.95).

## Discussion

Our findings demonstrate a significant positive association between the CONUT score and the severity of cerebrovascular stenosis. The key finding was that patients with cerebrovascular stenosis had significantly higher CONUT scores than those with normal vasculature, indicating a positive correlation between the CONUT score and the severity of intracranial and extracranial arterial stenosis. Stepwise regression further confirmed that a higher CONUT score is an independent risk factor for cerebrovascular stenosis. Notably, the CONUT score demonstrated moderate-to-good diagnostic performance (AUC = 0.794) with a sensitivity of 82.3% and specificity of 76.1% at the optimal cutoff (score ≥5) outperforming traditional risk factors such as age, diabetes, and all AUCs < 0.62, *p* < 0.001 (Table S1). Compared with individual components, the composite CONUT score improved specificity by 35% *versus* albumin alone and sensitivity by 24% *versus* lymphocyte count alone (Table S2). In addition, the study revealed an age-gradient effect in the predictive efficacy of the CONUT score: among individuals > 70 years, its predictive strength (OR = 3.15) was approximately twice that observed in younger adults (<50 years, OR = 1.82). Among patients with diabetes or hypertension, the risk increase (≈125%) did not differ statistically from that in healthy controls (111%) (Table S2). These findings indicate that the CONUT score retains stable predictive power across comorbid populations and support its utility as a cross-population screening tool—particularly for multimorbid elderly patients.

The association between the CONUT score and cerebrovascular stenosis may be underpinned by atherosclerosis, the primary aetiology of cerebrovascular stenosis. Notably, all three components of the CONUT score—albumin, total cholesterol, and lymphocyte count—are closely linked to the atherosclerotic process. This study also observed significant differences in these parameters between the stenosis and control groups.

**Albumin**: Human serum albumin is primarily synthesised in hepatocytes. Following processing in the endoplasmic reticulum and Golgi apparatus, it is released into the bloodstream as a mature protein (Zoanni et al., 2023). Albumin facilitates the transport of nutrients and drugs, regulates immune responses, and modulates cholesterol transport (Chien et al., 2017). Low albumin levels are associated with vascular endothelial dysfunction. Reduced albumin impairs erythrocyte deformability and increases blood viscosity, diminishing antioxidant capacity (Rozga, Piatek & Malkowski, 2013). This manifests as a weakened ability of albumin to scavenge oxygen free radicals in plasma and compromised vasodilatory effects (Lepedda et al., 2014). Hypoalbuminemia may also delay fibrinolysis, promote hypercoagulability, and potentially induce a prothrombotic state (Paar et al., 2017). Collectively, these factors may exacerbate atherosclerosis. further supports low albumin as an independent risk factor for cerebrovascular stenosis (Lin, Ke & Chen, 2023).

**Lymphocytes**: A reduced lymphocyte count may reflect a chronic inflammatory state, a key driver of atherosclerotic plaque development and instability (Björkegren & Lusis, 2022). Research indicates that B lymphocytes exhibit dual roles: B1 cells may confer atheroprotective effects, whilst B2 cells can promote atherosclerosis by enhancing pro-inflammatory cytokine production. Previous studies have linked lymphopenia (or related ratios such as Neutrophil-to-Lymphocyte Ratio (NLR) (Ying et al., 2021) and Monocyte-to-Lymphocyte Ratio (MLR) (Zuo et al., 2019)) to the severity of carotid or cerebrovascular stenosis (Anonymous, 2024).

**Cholesterol**: This study observed lower total cholesterol levels in the stenosis group, particularly in severe cases. This may reflect the overall poor nutritional status of patients within the CONUT framework. Cholesterol, ubiquitous in human cells, is integral to cell signalling, hormone synthesis, and metabolic homeostasis, and is implicated in the initiation and progression of inflammation (Zhou et al., 2023). Cholesterol exhibits a dual role in atherosclerosis: Elevated levels promote lipid deposition and foam cell formation; specifically, when low density liproprotein (LDL) infiltrates endothelial cells and undergoes oxidative modification, it triggers leukocyte aggregation, leading to endothelial damage and atherosclerotic plaque formation. Consequently, elevated LDL levels are associated with increased stroke risk (Ference et al., 2012; Turan et al., 2010). However, excessively low levels may also impair normal physiological functions, such as cell membrane stability. Hypocholesterolaemia often reflects chronic caloric deficiency and hepatic protein synthesis reprogramming within a chronic inflammatory state (Paraskevas et al., 2020). Paradoxically, a pronounced reduction in cholesterol may increase vascular fragility and compromise vascular stability. Multiple independent studies consistently identify very low LDL-C (<1.4 mmol/L), whether non-pharmacologically or pharmacologically induced, as an independent risk factor for intracerebral haemorrhage (ICH). For instance: The Japanese Jichi cohort found a 1.5- to 3.9-fold increased risk of haemorrhagic stroke mortality amongst individuals with low cholesterol (Okamura et al., 2024). A large Chinese real-world study (*n* = 794,000) confirmed that LDL-C <1.4 mmol/L was associated with a 26% higher risk of acute-phase Intracerebral Hemorrhage (ICH) (Lee & Hsu, 2025). Crucially, the lower total cholesterol observed in our stenosis group likely differs from pharmacologically induced reductions, as it co-occurs with hypoalbuminemia and lymphopenia. This pattern remains correlated with a malnutrition phenotype, which the CONUT score effectively captures as a multidimensional risk indicator. Naturally, the potential contribution of statin therapy to cholesterol lowering within our study population cannot be discounted. Future research should incorporate medication history and dynamic nutritional assessments to distinguish between pharmacologically mediated and nutritional depletion origins of hypocholesterolaemia.

The CONUT score incorporates albumin (nutrition and inflammation), lymphocyte count (immunity), and total cholesterol (lipid metabolism), aligning closely with the core pathological components of atherosclerotic vascular disease—nutrition, inflammation, immunity, and metabolism. Evidence of its predictive value in vascular disease: existing studies support the CONUT score’s predictive utility in cardiovascular contexts. For example: Li et al. (2025)’s study used the CONUT score to assess nutritional status in patients with moderate-to-severe aortic stenosis (AS), proving malnutrition as an independent risk factor for AS prognosis. Similarly, Di Vincenzo et al. (2023)’s research employed the CONUT score to evaluate malnutrition in AS patients and demonstrated its significant association with all-cause mortality. Son et al. (2023)’s study applied the CONUT score to assess nutritional status in carotid endarterectomy (CEA) patients, effectively identifying those at high risk of postoperative complications to guide clinical intervention. Arero et al. (2022) identified it as an independent predictor of adverse cardiovascular events in coronary heart disease patients; These supports CONUT’s applicability for vascular risk assessment. Regarding clinical operability, the CONUT score requires only routine hematological parameters, unlike the Geriatric Nutritional Risk Index (GNRI) (which necessitates height, weight, and ideal body weight calculations), making it simpler, faster, and better suited for routine clinical use. In summary, the CONUT score was selected for this study’s cerebrovascular stenosis risk assessment due to its comprehensive components, pathological relevance, established predictive evidence, and clinical practicality.

Furthermore, this study confirmed the association between traditional risk factors—including age, type 2 diabetes mellitus (T2DM), smoking history, and alcohol consumption—and cerebrovascular stenosis. These findings align with extensive prior research. For instance, elderly stroke patients exhibit a higher prevalence of symptomatic intracranial stenosis and poorer prognosis, with the prevalence of intracranial stenosis increasing with advancing age (Head, Daunert & Goldschmidt-Clermont, 2017). Diabetic patients demonstrate a higher prevalence of carotid atherosclerosis and plaque burden, and elevated fasting blood glucose levels correlate with greater stenosis severity (Katsiki & Mikhailidis, 2020). Nicotine and carbon monoxide in tobacco directly damage endothelial cells, elevate inflammatory mediators, increase coagulation factors, and heighten thrombotic risk (Kiriyama et al., 2020; Song et al., 2019). Studies indicate that excessive alcohol intake damages endothelial cells and promotes atherosclerosis, thereby increasing stroke risk (Krittanawong et al., 2022). Critically, these traditional factors were adjusted for as covariates in the statistical analyses. This further underscores the value of the CONUT score as an independent predictor of cerebrovascular stenosis.

## Conclusions

In conclusion, the CONUT score is positively correlated with the severity of cerebral vascular stenosis; higher scores indicate more severe stenosis. The CONUT score can serve as an independent risk factor for assessing the degree of cerebral vascular stenosis, offering predictive value and potential as an effective tool for screening and predicting the severity of cerebral vascular stenosis.

## Limitations

Several limitations of this study should be acknowledged. First, this study was a single-center, retrospective study with a limited sample size, which may introduce selection bias and restrict the generalizability of the findings. Second, due to the retrospective nature of this study, the influence of unknown confounding factors cannot be completely excluded. Third, although this study revealed a significant association between the CONUT score and cerebrovascular stenosis, establishing causality will require long-term follow-up investigations. Finally, the clinical utility of the CONUT score—such as whether improving nutritional status based on the CONUT score can effectively reduce the risk of stenosis or stroke incidence—remains to be confirmed through external validation and interventional studies. In addition, although the subgroup analysis showed that the CONUT score had significant predictive efficacy in the elderly population, the sample size of the group aged > 70 years was relatively small (*n* = 205). Future studies should focus on expanding the sample size, conducting prospective cohort studies, and evaluating the effectiveness of CONUT score-based intervention strategies, as well as validating the optimal cutoff value for this population in larger-scale studies.

##  Supplemental Information

10.7717/peerj.20968/supp-1 Supplemental Information 1 Original Data and Code

10.7717/peerj.20968/supp-2 Supplemental Information 2 Comparison of Predictive Performance of CONUT, Conventional Risk Factors, and Combined Models for Cerebrovascular Stenosis

10.7717/peerj.20968/supp-3 Supplemental Information 3 Individual components show limited predictive ability

## Additional Information and Declarations

### Data Availability

The following information was supplied regarding data availability: The raw measurements and code are available in the Supplemental File.
