# Supplementary material for: The relationship between controlling nutritional status (CONUT) and cerebrovascular stenosis: a retrospective study with implications for ischemic stroke prevention
Source: PeerJ. 2026 Mar 26;14:e20968. doi: 10.7717/peerj.20968 (PMC13033285; doi:10.7717/peerj.20968)
Supplement: Supplemental Information 1 [file peerj-14-20968-s001.zip › Supplement/Data Codebook.md]

# Data Codebook## Variables### 1. Gender- **Values**: 0, 1  - **Categories**:    - 0 = Female    - 1 = Male  - **Note**: This variable records the gender of participants using a binary coding system.### 2. CONUT Level (Nutritional Status)- **Values**: 1, 2, 3, 4  - **Categories**:    - 1 = Good nutritional status (all indicators within normal ranges)    - 2 = Mild malnutrition (some indicators slightly below normal)    - 3 = Moderate malnutrition (multiple indicators significantly below normal)    - 4 = Severe malnutrition (multiple indicators severely below normal, increasing the risk of complications and infections)  - **Note**: This variable assesses the nutritional status of participants, with higher values indicating more severe malnutrition.### 3. Hemadostenosis (Blood Viscosity Abnormality)- **Values**: 0, 1  - **Categories**:    - 0 = Present (disease state)    - 1 = Absent (non-disease state)  - **Note**: This variable records whether participants have blood viscosity abnormalities.### 4. Hypertension- **Values**: 0, 1  - **Categories**:    - 0 = Present (hypertension)    - 1 = Absent (no hypertension)  - **Note**: This variable records whether participants have hypertension.### 5. Diabetes- **Values**: 0, 1  - **Categories**:    - 0 = Present (diabetes)    - 1 = Absent (no diabetes)  - **Note**: This variable records whether participants have diabetes.### 6. History of Drinking- **Values**: 0, 1  - **Categories**:    - 0 = Present (history of alcohol consumption)    - 1 = Absent (no history of alcohol consumption)  - **Note**: This variable records whether participants have a history of alcohol consumption.### 7. Renal Insufficiency (Kidney Dysfunction)- **Values**: 0, 1  - **Categories**:    - 0 = Present (renal insufficiency)    - 1 = Absent (no renal insufficiency)  - **Note**: This variable records whether participants have renal insufficiency.### 8. CAD (Coronary Artery Disease)- **Values**: 0, 1  - **Categories**:    - 0 = Present (coronary artery disease)    - 1 = Absent (no coronary artery disease)  - **Note**: This variable records whether participants have coronary artery disease.### 9. History of Smoking- **Values**: 0, 1  - **Categories**:    - 0 = Present (history of smoking)    - 1 = Absent (no history of smoking)  - **Note**: This variable records whether participants have a history of smoking.
