# Supplementary material for: The relationship between controlling nutritional status (CONUT) and cerebrovascular stenosis: a retrospective study with implications for ischemic stroke prevention
Source: PeerJ. 2026 Mar 26;14:e20968. doi: 10.7717/peerj.20968 (PMC13033285; doi:10.7717/peerj.20968)
Supplement: Supplemental Information 2 [file peerj-14-20968-s002.docx]

**Supplementary Table S1** Comparison of Predictive Performance of CONUT, Conventional Risk Factors, and Combined Models for Cerebrovascular Stenosis

| **Variable** | **AUC** | **Sensitivity** | **Specificity** | ***p* value（VS CONUT）** |
| --- | --- | --- | --- | --- |
| CONUT | 0.794 | 0.766 | 0.809 | Reference |
| Age | 0.612 | 0.584 | 0.601 | <0.001 |
| Diabetes | 0.589 | 0.553 | 0.612 | <0.001 |
| Smoking | 0.621 | 0.598 | 0.627 | <0.001 |
| LDL-C | 0.531 | 0.492 | 0.567 | <0.001 |
| CONUT+Age+HDL | 0.832 | 0.801 | 0.822 | 0.013 |

CONUT is the strongest single predictor: AUC = 0.794 (95% CI: 0.765-0.822), significantly higher than: Age (AUC=0.612, ΔAUC=-0.182, p<0.001) ；Diabetes (AUC=0.589, ΔAUC=-0.205, p<0.001) ；LDL-C (AUC=0.531, ΔAUC=-0.263, p<0.001) ；(Full comparison in new Supplementary Table S1)

Limited added value from combining factors: The optimal combination was CONUT + Age + HDL-C: AUC = 0.832 (95% CI: 0.806-0.858) ；Net reclassification improvement (NRI) = 0.12 (p=0.03)；This represents only a modest 4.8% improvement over CONUT alone；Simplicity vs benefit analysis favors standalone CONUT in screening

CONUT integrates three critical pathways linked to stenosis pathogenesis: albumin (endothelial dysfunction + oxidative stress), lymphocytes (chronic vascular inflammation), and cholesterol (plaque vulnerability). CONUT outperformed conventional risk factors in stenosis prediction. Though combinatory models slightly improved accuracy, the marginal gain may not justify added complexity in clinical practice.
