# Supplementary material for: The relationship between controlling nutritional status (CONUT) and cerebrovascular stenosis: a retrospective study with implications for ischemic stroke prevention
Source: PeerJ. 2026 Mar 26;14:e20968. doi: 10.7717/peerj.20968 (PMC13033285; doi:10.7717/peerj.20968)
Supplement: Supplemental Information 3 [file peerj-14-20968-s003.docx]

| **Component** | **AUC** | **Optimal Threshold** | **Major Limitation** |
| --- | --- | --- | --- |
| Albumin | 0.646 | <38 g/L | Low specificity: 58% of hypoalbuminemia cases were due to non-vascular causes (liver/kidney disease) |
| Cholesterol | 0.531 | <4.1 mmol/L | Poor discrimination: 62% of low-cholesterol cases had no stenosis |
| Lymphocyte | 0.572 | <1.8×10⁹/L | Low sensitivity: 45% of stenosis patients had normal counts |

**Supplementary Table S2** Individual components show limited predictive ability

| **Predictive Indicator** | **AUC** | **Sensitivity** | **Specificity** | **PPV** | **NPV** |
| --- | --- | --- | --- | --- | --- |
| Albumin | 0.646 | 0.621 | 0.598 | 0.68 | 0.54 |
| Lymphocytes | 0.572 | 0.55 | 0.562 | 0.63 | 0.48 |
| Cholesterol | 0.531 | 0.502 | 0.543 | 0.59 | 0.45 |
| CONUT | 0.794 | 0.766 | 0.809 | 0.83 | 0.74 |

Composite CONUT achieves superior performance: Specificity increased by 35% (80.9% vs ≤59.8% for components)，Sensitivity increased by 24% (76.6% vs ≤62.1%)

Clinical consequence of using composite score: In our cohort, CONUT would: Avoid 142 unnecessary DSA referrals vs albumin-based screening (calculated: 383 controls × (1-specificity difference)) Miss 23 fewer true stenosis cases vs lymphocyte-based screening
